# Augmented Reality-Assisted Placement of Surgical Guides and Osteotomy Execution for Pelvic Tumour Resections: A Pre-Clinical Feasibility Study Using 3D-Printed Models

**DOI:** 10.3390/cancers17132260

**Published:** 2025-07-07

**Authors:** Tanya Fernández-Fernández, Javier Orozco-Martínez, Amaia Iribar-Zabala, Elena Aguilera Jiménez, Carla de Gregorio-Bermejo, Lydia Mediavilla-Santos, Javier Pascau, Mónica García-Sevilla, Rubén Pérez-Mañanes, Jose Antonio Calvo-Haro

**Affiliations:** 1 Department of Orthopaedic Surgery and Traumatology—Musculoskeletal Oncology Division, Hospital General Universitario Gregorio Marañón, Dr. Esquerdo 46, 28007 Madrid, Spain; javier.orozco@salud.madrid.org (J.O.-M.); lydia.mediavilla@salud.madrid.org (L.M.-S.); joseantonio.calvo@salud.madrid.org (J.A.C.-H.); 2 Digital Health and Biomedical Technologies, Vicomtech Foundation, Basque Research and Technology Alliance (BRTA), 20009 Donostia-San Sebastian, Spain; airibar@vicomtech.org; 3 Advanced Planning and 3D Manufacturing Unit (UPAM3D), Hospital General Universitario Gregorio Marañón, Dr. Esquerdo 46, 28007 Madrid, Spain; elena.aguilera.externo@salud.madrid.org (E.A.J.); carla.gregorio@salud.madrid.org (C.d.G.-B.); ruben.perez@salud.madrid.org (R.P.-M.); 4 Bioengineering Department, Universidad Carlos III de Madrid, 28911 Leganés, Spain; jpascau@ing.uc3m.es (J.P.); mongarci@ing.uc3m.es (M.G.-S.)

**Keywords:** augmented reality (AR), patient-specific instruments (PSI), head-mounted display (HMD), 3D-printed guides, osteotomy accuracy

## Abstract

**Objectives**: This pre-clinical feasibility study evaluates the accuracy of a novel augmented reality-based (AR-based) guidance technology using head-mounted displays (HMDs) for the placement of patient-specific instruments (PSIs)—also referred to as surgical guides—and osteotomy performance in pelvic tumour resections. The goal is to improve PSI placement accuracy and osteotomy execution while assessing user perception and workflow efficiency. **Methods**: The study was conducted on ten 3D-printed pelvic phantoms derived from CT scans of cadaveric specimens. Custom PSIs were designed and printed to guide osteotomies at the supraacetabular, symphysial, and ischial regions. An AR application was developed for the HoloLens 2 HMD to display PSI location and cutting planes. The workflow included manual supraacetabular PSI placement, AR-guided placement of the other PSIs and osteotomy execution. Postoperative CT scans were analysed to measure angular and distance errors in PSI placement and osteotomies. Task times and user feedback were also recorded. **Results**: The mean angular deviation for PSI placement was 2.20°, with a mean distance error of 1.19 mm (95% CI: 0.86 to 1.52 mm). Osteotomies showed an overall mean angular deviation of 3.73° compared to planned cuts, all within the predefined threshold of less than 5°. AR-assisted guidance added less than two minutes per procedure. User feedback highlighted the intuitive interface and high usability, especially for visualising cutting planes. **Conclusions**: Integrating AR through HMDs is a feasible and accurate method for enhancing PSI placement and osteotomy performance in pelvic tumour resections. The system provides reliable guidance even in cases of PSI failure and adds minimal time to the surgical workflow while significantly improving accuracy. Further validation in cadaveric models is needed to ensure its clinical applicability.

## Simple Summary

This study explores a novel augmented reality system integrated with head-mounted displays to assist pelvic tumour resections. The technology offers dual functionality: it aids in the accurate placement of patient-specific surgical guides and displays cutting planes directly in the surgeon’s field of view to enhance osteotomy execution. Tested on 3D-printed pelvic models, the system achieved high precision, with less than 3 degrees of angular deviation and under 2 mm of distance error for guide placement. Osteotomies closely matched planned cuts, staying within a 5-degree threshold. The system maintained reliability even when physical guides failed, as the holographic display continued to provide visual reference. Surgeons reported the interface as intuitive and effective, particularly for visualising cutting planes. The procedure required less than two additional minutes, supporting its efficiency. These results demonstrate the feasibility of this approach for improving surgical accuracy, with further validation on cadaveric models needed before clinical application.

## 1. Introduction

Freehand tumour resections often fail to achieve adequate surgical margins, with a success rate of only 52% (95% CI: 37–67) when using conventional techniques [1]. To address this, various technologies have been developed to assist surgeons, including computer-assisted navigation based on optical tracking systems (OTS) [2,3], patient-specific instruments (PSIs) [4,5,6] and augmented reality (AR) [7,8,9,10,11,12].

Navigation systems offer real-time tracking of surgical instruments using preoperative imaging. However, these systems require a clear line of sight between tracking markers and cameras, which can be challenging to maintain in surgical environments. Additionally, navigation setups are cumbersome and require significant intraoperative preparation time, limiting their practicality [4].

PSIs offer predefined cutting paths specific to the patient’s anatomy, facilitating accurate osteotomies [13]. They are widely used due to their convenience and precision in different surgical scenarios, especially in oncological procedures [5]. Recent clinical analyses have demonstrated that PSIs not only increase the rate of achieving tumour-free margins but also contribute to improved relapse-free and overall survival rates [14]. However, inadequate surface matching, variations in guide design and the presence of soft tissue on the skeletal surface can complicate the accurate placement of PSIs, potentially reducing their accuracy or introducing errors. Additionally, homogenous and smooth bone regions pose significant challenges for manual PSI placement, increasing the likelihood of misalignment and higher error rates.

AR technology has emerged as a promising tool to enhance surgical accuracy by overlaying virtual elements onto the surgeon’s field of view [9,10]. AR integrates virtual sensory impressions, such as anatomical structures and cutting planes, into the surgeon’s real-world view, providing real-time guidance directly on the surgical site and enhancing precision without requiring separate monitors. A unique advantage of AR is its ability to display osteotomy planes directly on the surgical site, providing intuitive guidance. Hoch et al. recently showed promising results integrating AR in peri-acetabular osteotomies [3,15]. However, to our knowledge, no studies have directly demonstrated its impact on improving pelvic tumour osteotomy accuracy, making this a key focus of our research.

This capability can be implemented using various devices, including smartphones, tablets or head-mounted displays (HMDs) [16]. Smartphone- or tablet-based AR systems are portable and affordable but require the surgeon to hold the device, limiting dexterity. In contrast, HMDs offer a hands-free solution, allowing continuous visualisation from the surgeon’s point of view. The study by García-Sevilla et al. demonstrated that integrating AR with PSI placement results in greater accuracy compared to conventional manual placement, reducing angular deviations and improving osteotomy precision [13]. Although their proof-of-concept study was limited to two phantoms, it provides a foundational basis for further research, including ours. We believe that HMDs offer a more practical and comfortable solution for surgeons in real-life surgical settings, allowing hands-free visualisation and reducing the need to look away from the operative field.

This study aims to validate the feasibility and accuracy of integrating AR based on HMDs with personalised 3D-printed PSIs for guiding complex pelvic osteotomies by assisting in PSI placement and visually displaying the osteotomy planes. The proposed tool is designed to apply not only to pelvic osteotomies but also to other anatomical regions requiring precise bone resections, making it a versatile solution for various surgical scenarios.

We hypothesise that integrating AR through an HMD to guide both PSI placement and osteotomy performance can enhance surgical accuracy in pelvic tumour resections. This study builds upon a recent proof-of-concept investigation by our research group, which demonstrated the feasibility of an AR-guided surgical workflow using phantom models derived from cadaveric CT segmentations [17]. That preliminary work, conducted by Iribar-Zabala et al., focused on validating the technical methodology, including workflow design and accuracy evaluation, within a controlled experimental setting. In a more clinically oriented context, building on these findings, our study is among the first to evaluate the effectiveness of AR in guiding both PSI placement and osteotomy execution using HMDs, focusing on achieving optimal accuracy in a pre-clinical setting. Based on the generally accepted tolerance for osteotomies [8,9,10,12,18], we define optimal PSI placement accuracy as an angular deviation of less than 3° and a mean distance error of less than 2 mm. For osteotomy accuracy, optimal performance is defined as an overall angular deviation of less than 5°.

This pre-clinical feasibility study uses 3D-printed pelvic phantoms to evaluate the accuracy of the AR-based guidance system and identify potential areas for improvement. Subsequent phases of this research will involve cadaveric models for further clinical validation.

## 2. Materials and Methods

A computed tomography (CT) scan with a 512 × 512 matrix and a pixel size of 0.98 mm was acquired for ten cadaveric specimens. In all cases, the left hemipelvis was arbitrarily chosen to avoid any bias related to surgeon preference for size or side.

**Figure 1:**
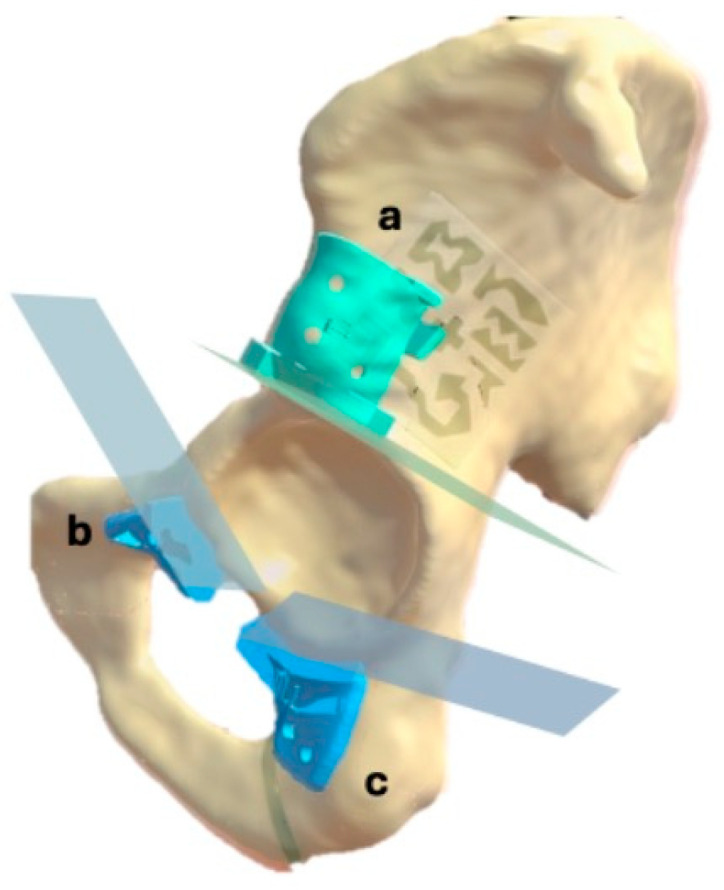
Segmented left hemipelvis showing the three designed PSIs and their corresponding planned cutting planes: supraacetabular (**a**), symphysial (**b**) and ischial (**c**).

The bone structures of the hemipelvis were manually segmented using 3D Slicer software (version 5.6.2, The Slicer Community, Brigham and Women’s Hospital, Boston, MA, USA). Based on this segmentation, an expert clinician defined three cutting planes in the supraacetabular, ischial and symphysial regions to achieve acetabular resection. These cutting planes served as a reference for designing three patient-specific instruments (PSIs) to indicate the cutting planes during the procedure (Figure 1). The PSIs were designed using 3-matic software (version 16.0, Materialise NV, Leuven, Belgium), with holes included for fixation to the bone using 3D-printed pins.

**Figure 2:**
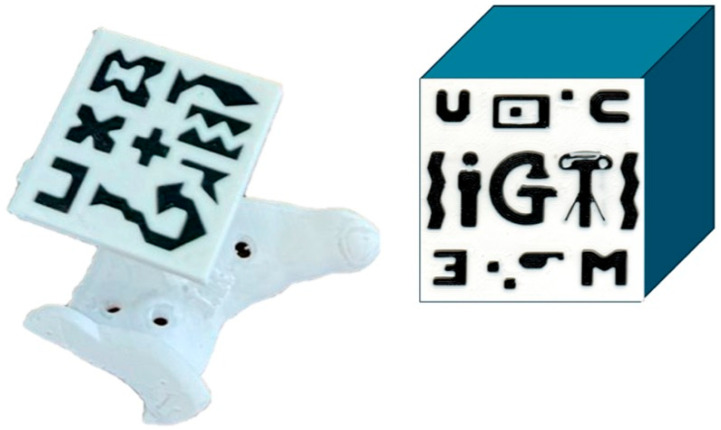
The 4 × 4 cm fiducial marker for AR detection. On the (**left**), the designed version for the supraacetabular PSI socket. On the (**right**), the unique recognisable pattern of the marker complies with Vuforia’s standards for marker detection.

A socket was integrated into the supraacetabular PSI to accommodate an augmented reality (AR) marker, designed to meet Vuforia’s quality standards for marker detection [19]. The decision to allocate the AR marker to the supraacetabular PSI was based on findings from García-Sevilla et al., demonstrating that this region is associated with the least deviation error during manual placement [6]. The marker measured 4 × 4 cm and featured a unique, recognisable pattern (Figure 2).

**Figure 3:**
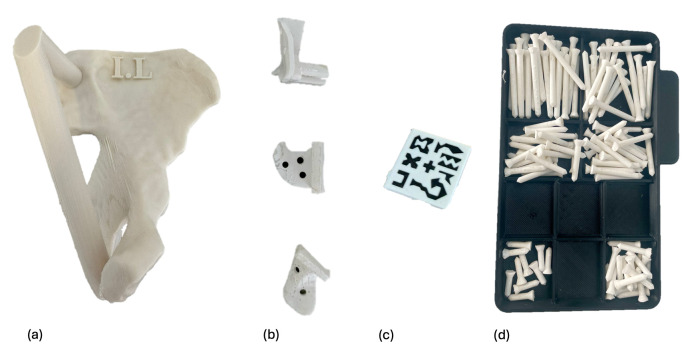
All components were 3D-printed. Each phantom (**a**) was printed in ASA and identified with a label, such as I.L (I in Roman numerals for specimen 1, and L for left). The PSIs (**b**) and fixation pins (**d**) were printed in rigid 10 K resin. The fiducial AR marker (**c**) was printed in PLA.

Components were 3D printed using different materials and printers (Figure 3). The healthy bone portions were printed with acrylonitrile styrene acrylate (ASA) material, while the AR markers were printed with a dual extruder using black and white polylactic acid (PLA) filament on a Bambu Lab X1E printer (Bambu Lab, Shenzhen, China). The PSIs were designed with at least three holes for a 3.2 mm drill, with a tolerance of 0.6 mm, to accommodate fixation pins with a 3.5 mm diameter. Both the PSIs and fixation pins were printed using rigid 10K resin, a radiopaque material, with a Form 2 printer (Formlabs, Somerville, MA, USA). The selection of these materials aimed to facilitate post-experiment segmentation from CT scans.

An AR application was developed for the HoloLens 2 head-mounted display (HMD) (Microsoft Corporation, Redmond, WA, USA) to assist with navigation throughout the workflow of PSI placement. The application was created using the Unity engine, integrating the Mixed Reality Toolkit (MRTK) for enhanced functionality and user interaction.

**Figure 4:**
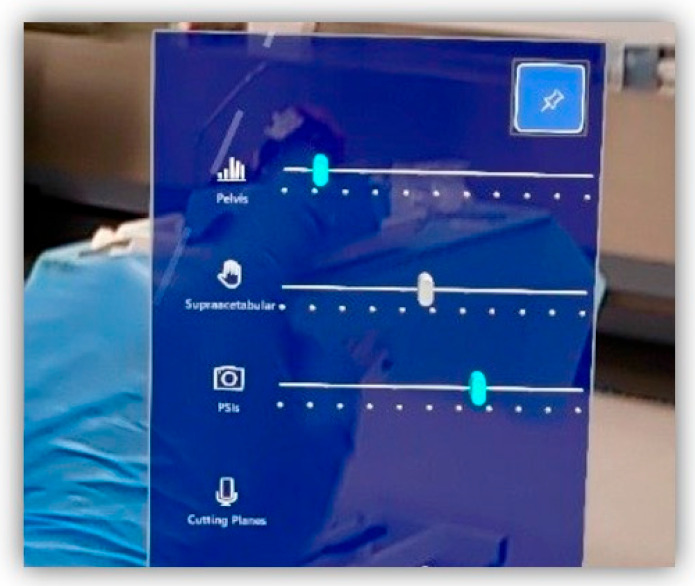
Custom-designed Vuforia menu from our software, allowing the user to select features to display (bone, supraacetabular PSI, remaining PSIs and cutting planes) and adjust transparency levels for better visualisation.

**Figure 5:**
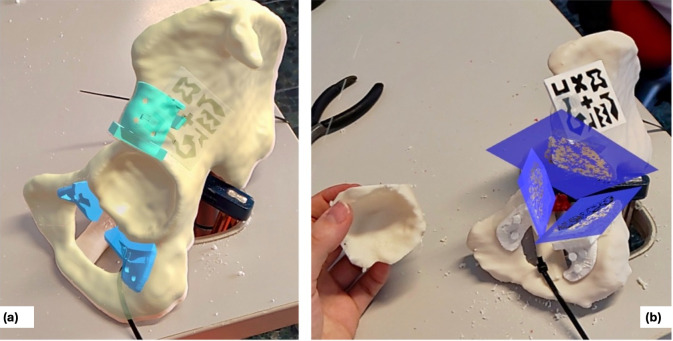
Captured images from the surgeon’s view through the HMDs (AR). (**a**) shows the suggested positions for the symphysial and ischial PSIs (sky blue) after supraacetabular placement (turquoise). (**b**) shows the isolated cutting planes (dark blue) following osteotomy execution.

Precise hologram positioning was achieved using the Vuforia library, which allowed for accurate marker detection. The application featured a hand menu with on/off buttons to visualise PSIs relative to the AR marker placed on the supraacetabular region, as well as sliders to adjust the transparency of both the PSIs and the bone (Figure 4). Cutting planes were also displayed with on/off buttons for additional guidance during osteotomy execution (Figure 5).

**Figure 6:**
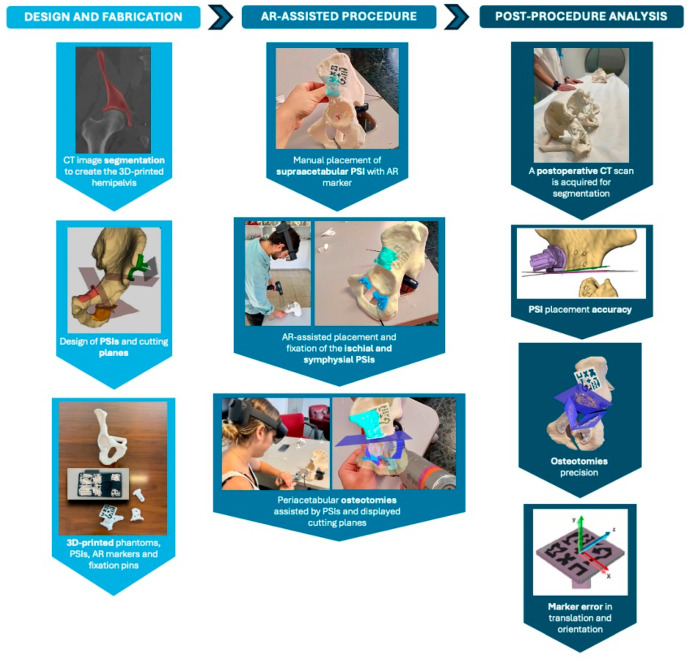
Workflow of the experiment and surgical scenario, organised into three phases: design and fabrication, AR-assisted procedure and post-procedure analysis.

The comprehensive workflow, illustrated in Figure 6, included three main phases: design and fabrication, AR-assisted procedure and post-procedure analysis. The initial step involved acquiring the CT scan, segmenting the bone and designing the PSIs based on the defined cutting planes. Once all components were fabricated, two expert clinicians performed the experimental procedure, with one clinician handling odd-numbered cases and the other clinician managing even-numbered cases, following these steps:

1. Manual placement and fixation of the supraacetabular PSI.
2. Placement of the AR marker on the supraacetabular PSI.
3. AR-assisted placement and fixation of the symphysial and ischial PSIs using the hologram displayed on the HoloLens 2.
4. Peri-acetabular osteotomies guided by the cutting planes displayed through the HoloLens 2 and supported by the positioned 3D-printed guides.

After the procedure, a postoperative CT scan was acquired for each phantom. All structures, including the PSIs and the bone, were segmented to evaluate the positional deviation of the PSIs, the accuracy of the osteotomies and the error introduced by the AR marker. The same workflow will be applied in future cadaveric studies and, potentially, in patient procedures. For clinical applications, PSIs will be removed after surgery, and analysis will be adapted accordingly.

The evaluation metrics for this study focused on five key aspects: PSI placement accuracy, osteotomy precision, fiducial marker error, procedure duration and user perception and satisfaction.

PSI placement accuracy was assessed by comparing the planned and final planes derived from the PSIs. Angular deviation between these planes was calculated by measuring the angle between their normal vectors. Specifically, let n_1_ and n_2_ represent the normal vectors of the planned and final planes, respectively, with the angle θ between the planes computed using the cross product. Additionally, maximum distance deviation was determined using the iterative closest point (ICP) algorithm to match corresponding points on the planned and final surfaces, providing mean values with a 95% confidence interval and maximum distance errors.

**Figure 7:**
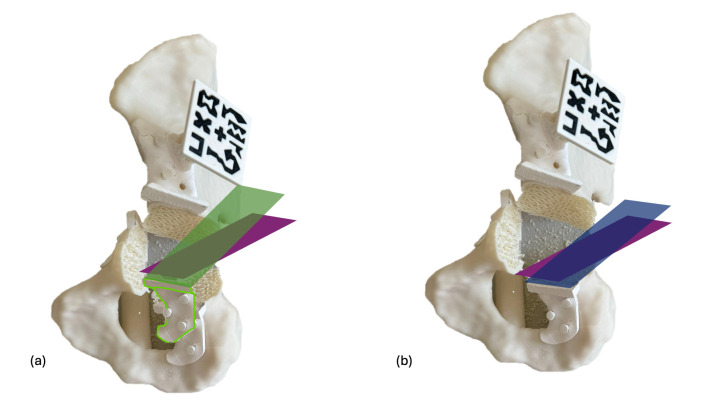
Osteotomy deviation is evaluated in two scenarios. In the first scenario (**a**), the final cut (purple) is compared to the initially designed cutting plane (green), noting a slight deviation in the PSI placement (white) compared to the planned position (green shape). In the second scenario (**b**), the angular deviation of the performed osteotomy (purple) is measured against the cutting plane suggested by the PSI after placement (blue), which should align with the plane visualised through the HMD if the PSI is correctly positioned.

Osteotomy accuracy was evaluated through postoperative CT scans, conducting two analyses: comparison of the final osteotomy cuts with the initial planned cuts, and comparison with the displayed cutting planes after positioning the supraacetabular PSI (Figure 7). The overall angular deviation of the final osteotomy was calculated by comparing the computed planes with the actual cuts in both scenarios. A Wilcoxon signed-rank test was used to detect significant differences between the two groups, and a Bland–Altman plot analysis was generated to assess agreement between measurements.

The AR marker error was measured by comparing preoperative and postoperative CT scans to identify discrepancies in the marker’s position. Translation and rotation errors were calculated relative to the supraacetabular marker and decomposed into roll, pitch, and yaw angles. Translation and rotation errors were calculated by registering pre- and postoperative CT scans, computing the transformation matrix between planned and actual marker positions, and decomposing the resulting differences relative to a marker-centred coordinate system, as detailed in our previously published methodology [17].

Task time was recorded for each procedural step, including the placement and fixation of the supraacetabular, symphysial and ischial PSIs, as well as the execution of the corresponding osteotomies.

User perception and satisfaction were assessed to evaluate the practical application of the AR-assisted workflow. After completing the procedure, clinicians provided subjective feedback on the system’s usability, clarity of holographic guidance and overall satisfaction. Key aspects evaluated included the intuitiveness of the AR interface, the accuracy of visual guidance and the perceived impact on procedural efficiency and confidence during osteotomy execution.

## 3. Results

The following sections show the results for ten phantoms from different cadavers. Each model is named in Roman numbers according to their identification with the cadaver specimen.

### 3.1. PSI Placement Accuracy

Table 1 and Table 2 present the detailed angular error (°) and mean and maximum distance deviations (mm) for each PSI placement and corresponding regional mean values, respectively. All PSIs demonstrated low angular errors, with an overall mean value of 2.20° ± 2.41°, with a 95% confidence interval (CI) of 1.28° to 3.11°. The supraacetabular PSI showed the least angular deviation, with a mean error of 1.44° ± 1.13°. The mean distance error was 1.19 ± 0.53 mm, with a 95% CI of 0.86 to 1.52 mm. The supraacetabular region exhibited the most consistent placement accuracy, while the ischial and symphysial regions showed higher variability, although all deviations remained within the targeted thresholds.

### 3.2. Osteotomy Precision Analysis

Table 3 presents the detailed mean angular deviation of the performed osteotomies compared to both the initial planned osteotomy and the suggested cutting plane after PSI placement. The supraacetabular osteotomy shows the least angular error. The mean overall angular error is 3.73° when compared to the planned osteotomy and 3.54° when compared to the suggested osteotomy after PSI placement (Table 4). Both values are within the targeted threshold (<5°), with 95% confidence intervals (CI) of (1.79, 4.40) and (1.76, 4.32), respectively.

**Figure 8:**
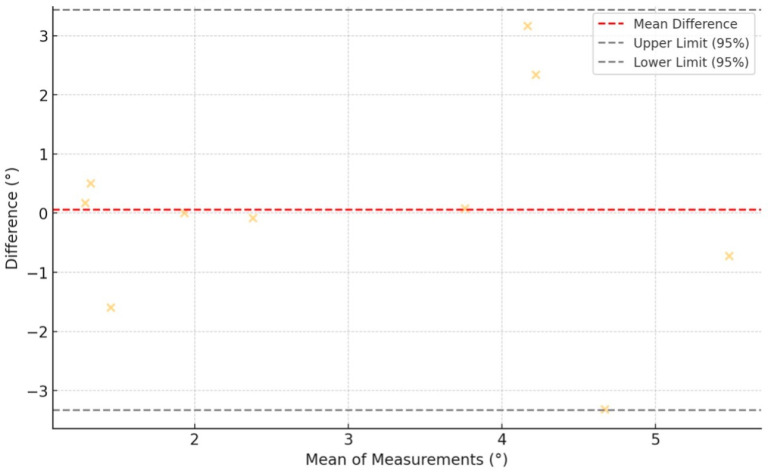
Bland–Altman plot showing agreement between angular osteotomy deviations from the initially planned planes and the PSI-suggested planes. Yellow crosses represent individual measurement differences plotted against their means. The red dashed line indicates the mean difference (bias), while the black dashed lines show the 95% limits of agreement. Most points fall within these limits, suggesting good agreement between methods.

A comparative analysis between both groups was conducted using the Wilcoxon signed-rank test, revealing no significant statistical differences (*p* = 0.846). The Bland–Altman plot (Figure 8) comparing the angular deviations from the initially planned osteotomy and the suggested osteotomy by the placed PSIs shows that the differences between the two measurements were generally small and fell within the 95% limits of agreement, indicating good consistency between the two sets of deviations.

### 3.3. Fiducial Marker Error

The following data (Table 5) shows the error associated with the supraacetabular AR marker. In general, the obtained values are low, below 1 mm in translation and 1° in rotation.

### 3.4. Procedure Duration

Table 6 presents the average times and standard deviations for PSI placement and fixation and the times recorded for osteotomy execution. The combined time for PSI placement and fixation adds an average of 113.88 s to the overall surgical workflow.

### 3.5. User Perception and Satisfaction

The two users involved in the experiment provided subjective feedback on the AR-assisted workflow. The system was rated 3/5 for ease of learning, with positive scores for software interface design and HMD ergonomics (4/5 each). AR adaptation and image quality were highly rated, with a score of 5/5 for both. Users highlighted the significant clinical value added to the operating room workflow for both assisting with the PSI placement and especially for the cutting planes display.

Both recommended broader use of the technology but noted two areas for improvement: depth offset in the AR display (rated 3/5) and button interaction precision. Despite these issues, users adapted quickly and found the system beneficial.

## 4. Discussion

The primary goal of this study was to minimise errors in patient-specific instrument (PSI) placement and osteotomy performance in pelvic tumour resections by integrating augmented reality (AR) technology. Various AR display devices, including smartphones, tablets and head-mounted displays (HMDs), are available for surgical use. Each has distinct advantages and drawbacks. Smartphone-based AR systems, as described by Moreta et al., are more affordable and practical, allowing visualisation through sterile bags or cases like CleanCase (Steridev Inc., Lansing, MI, USA) [16,20]. However, smartphones require the surgeon to hold the device, compromising dexterity and workflow efficiency. In contrast, HMDs, such as the HoloLens 2 used in our study, offer hands-free visualisation directly within the surgeon’s field of view. Despite the higher cost and potential discomfort with prolonged use [21], we opted for the HMD approach due to its ability to provide continuous, intuitive guidance without interrupting the surgical process. This choice enhances the practicality of the system in real surgical environments by reducing the need for surgeons to look away from the operative field, thus improving workflow efficiency and minimising potential distractions.

Our study demonstrates that integrating AR with HMDs achieves the intended goal of enhancing accuracy in PSI placement and osteotomy execution. The mean angular deviation for PSI placement was 2.20°, with a mean distance error of 1.19 mm (95% CI: 0.86 to 1.52 mm), well within the predefined accuracy thresholds. Previous studies by Sallent et al. and Mediavilla et al. have shown that using PSIs significantly improves osteotomy accuracy compared to free-hand techniques [5,14,22]. Our results demonstrate that further assisting PSI placement with AR can achieve even greater precision, reducing angular and distance errors. Similarly, a recent study by Hoch et al. reported higher deviation rates in Ganz pelvic osteotomies using AR-guided PSIs, with mean angular errors ranging from 6° to 7° and mean distance deviations around 9 mm [15]. Our findings show a marked improvement over these values, emphasising the potential of AR integration to enhance surgical precision in complex pelvic resections.

A similar scenario was reported by Ogawa et al., who developed the AR-HIP system, an augmented reality device for acetabular cup placement. They found absolute differences in cup inclination and anteversion angles of 2.1° ± 1.5° and 2.7° ± 1.7°, respectively, in a pilot study [23]. Tsukada et al. reported similar results with differences of 2.5° ± 1.7° and 2.1° ± 1.8°, respectively [24]. Kimura et al. further explored a pin-less AR application for acetabular placement and obtained comparable results, with the percentage of acetabular cups placed within ±5° of the target angles significantly higher in the pin-less AR navigation group (90.3%) [7]. While these studies focused on acetabular placement in total hip arthroplasty, they highlight the growing use of AR technologies in pelvic surgeries. These results, though in non-oncological contexts, demonstrate the replicability of AR-assisted workflows in achieving high accuracy in pelvic bone procedures. In the oncological setting, Wang et al. demonstrated that tumour osteotomies performed with fluoroscopically calibrated PSIs achieved mean distance errors of 2.66 mm and mean angular deviations of 2.16° [25]. These promising results align with our findings. However, our approach offers the distinct advantage of visualising cutting planes directly in the surgeon’s field of view through HMDs, eliminating the need for intermittent fluoroscopic guidance and reducing radiation exposure.

During our experimental procedures, several technical challenges arose that could have impacted accuracy but ultimately highlighted the robustness of the proposed workflow. In Case VII, the supraacetabular PSI’s marker socket broke during the symphysial osteotomy. Despite this setback, the software’s static guidance feature allowed us to complete the osteotomy and the subsequent ischial osteotomy without significant deviations. This scenario underscores a critical advantage of AR-based systems—the ability to continue guiding osteotomies accurately even if the physical PSI is compromised. Such resilience can be particularly valuable in real-life surgical workflows, where unexpected disruptions may occur.

Another challenge occurred in Case II, where the phantom support broke completely during the symphysial osteotomy, affecting the pubic branch. This structural failure prevented the analysis of the symphysial PSI placement for that case. Additionally, in Cases I, II, and X, the symphysial PSI broke during fixation, likely due to its smaller size and the stress concentrated around the holes. Despite these issues, the final osteotomies in these cases did not show significant deviations, suggesting that AR-assisted visualisation played a compensatory role. It is essential to have the supraacetabular PSI, which houses the AR marker, accurately placed because it provides the reference for precise hologram visualisation [6,13,17]. Even if a PSI breaks, the cutting planes displayed by the HMD allow surgeons to perform osteotomies hands-free, following the AR guidance to ensure accuracy. This feature enhances surgical safety and workflow resilience, allowing procedures to continue seamlessly even in the event of equipment failure. This added-value feature is crucial in clinical scenarios where PSIs may fail or become compromised. The AR system provides a secondary verification tool to validate or adjust the planned osteotomy planes, ensuring accuracy throughout the procedure.

Our statistical analysis also supports the reliability of the AR-assisted workflow. The Wilcoxon signed-rank test comparing the final executed osteotomy planes with both the initial planned cuts and the suggested cuts after AR-assisted PSI placement showed no significant differences (*p* = 0.846). This finding indicates that the AR system effectively maintains accuracy and prevents cumulative errors during the workflow. The results demonstrate that the AR-guided PSI placement achieves accurate osteotomies without introducing significant deviations, ensuring that each step of the process is reliable and reproducible.

Among the three osteotomy planes, the ischial osteotomy showed the highest error rates. This discrepancy is likely due to the order of osteotomy execution in our proposed workflow. As each osteotomy is performed, the structural stability of the phantom is progressively reduced, particularly after the supraacetabular and symphysial cuts. Additionally, the acrylonitrile styrene acrylate (ASA) material used to print the models exhibits a degree of flexibility that may have contributed to increased deviations during the ischial osteotomy. However, the AR system compensates for these limitations by providing precise guidance, adding less than two minutes to the surgical workflow. User feedback highlighted the system’s intuitive cutting plane display and transparency adjustment features, which enhanced confidence and precision during osteotomies. Clinicians quickly adapted to the system, reporting improved visualisation and workflow efficiency despite minor issues with depth perception and button interaction.

The choice of phantoms for this study was appropriate for feasibility analysis, as they allowed us to evaluate the system’s accuracy in a controlled environment. Previous studies, such as those by Olexa et al., have demonstrated the value of using phantoms for preclinical assessments before transitioning to cadaveric models [26]. However, the limitations of phantom models must be acknowledged. Phantoms lack soft tissues, intraoperative bleeding and do not fully replicate the mechanical properties of human bone, which may influence the accuracy of PSI placement and osteotomy performance. Despite these limitations, our results provide a solid foundation for further validation in cadaveric studies.

Another limitation of our study is the relatively small sample size of ten specimens. While this number is sufficient for a proof-of-concept study, larger sample sizes will be needed in future experiments to confirm the reproducibility and generalizability of our findings. Additionally, future research should focus on optimising the AR interface and addressing the minor usability issues identified by clinicians to further improve the system’s practical application in surgical settings.

## 5. Conclusions

In conclusion, our study demonstrates the feasibility of this innovative technology, highlighting its precision and user acceptance. Integrating AR through HMDs achieves accurate PSI placement and osteotomy execution in complex pelvic resections, with mean angular deviation and distance error rates within acceptable thresholds. The accuracy and reliability of AR guidance, even in cases of PSI failure, underscore its added value in real-life workflows. User feedback highlights the system’s intuitive visualisation of cutting planes as a key advantage. Further clinical experiments involving cadaveric models are essential to validate its performance in realistic surgical scenarios.

## Figures and Tables

**Table 1:** Angular and distance errors in PSI placement.

Case	PSI	Angular Error (°)	Mean Distance Error (mm)
I	Supraacetabular	0.58	1.49 ± 0.23
	Symphysial	3.65	1.15 ± 0.79
	Ischial	1.64	1.51 ± 1.02
II	Supraacetabular	2.55	1.64 ± 0.63
	Symphysial	NA ^1^	NA ^1^
	Ischial	9.05	3.66 ± 1.90
III	Supraacetabular	0.94	1.72 ± 0.29
	Symphysial	0.67	0.59 ± 0.23
	Ischial	2.96	1.97 ± 0.72
IV	Supraacetabular	0.47	0.13 ± 0.01
	Symphysial	0.07	0.09 ± 0.32
	Ischial	0.82	0.54 ± 0.40
V	Supraacetabular	0.01	1.35 ± 0.01
	Symphysial	1.13	0.90 ± 0.20
	Ischial	2.28	0.73 ± 0.63
VI	Supraacetabular	2.08	0.92 ± 0.42
	Symphysial	1.73	0.89 ± 0.24
	Ischial	1.29	0.71 ± 0.56
VII	Supraacetabular	3.79	2.61 ± 1.13
	Symphysial	1.67	0.53 ± 0.27
	Ischial	1.67	0.41 ± 0.35
VIII	Supraacetabular	0.89	2.64 ± 0.27
	Symphysial	1.83	1.16 ± 0.32
	Ischial	1.38	0.54 ± 0.51
IX	Supraacetabular	1.75	0.51 ± 0.31
	Symphysial	1.28	0.59 ± 0.33
	Ischial	2.09	0.36 ± 0.30
X	Supraacetabular	1.33	0.93 ± 0.45
	Symphysial	11.27	2.54 ± 1.69
	Ischial	2.83	1.83 ± 0.83

^1^ Data not available for Case II (Symphysial PSI placement) due to structural failure of the phantom. The support broke completely during the symphysial osteotomy, compromising the pubic branch and preventing reliable analysis.

**Table 2:** Mean regional and overall angular deviations in PSI placement.

PSI	Angular Error (°)	CI (95%) Angular Deviation	Mean Distance Error (mm)	CI (95%) Distance Deviation	Mean Max. Distance Error (mm)
Supraacetabular	1.44 ± 1.13	(0.63, 2.25)	1.39 ± 0.38	(1.16, 1.62)	2.54
Symphysial	2.59 ± 3.40	(0.03, 5.20)	0.94 ± 0.49	(0.64, 1.24)	2.17
Ischial	2.60 ± 2.36	(0.91, 4.29)	1.23 ± 0.72	(0.78, 1.68)	3.68
Total	2.20 ± 2.41	(1.28, 3.11)	1.19 ± 0.53	(0.86, 1.52)	2.82

**Table 3:** The detailed mean angular error of the final osteotomy planes compared to the originally planned osteotomy and the suggested osteotomy after PSI placement, respectively.

Case	Osteotomy	FINAL Cut vs. PLANNED Cut Mean Angular Error (°)	FINAL Cut vs. PLACED PSI SUGGESTED Cut Mean Angular Error (°)
I	Supraacetabular	1.58	1.07
	Symphysial	4.71	2.58
	Ischial	3.01	2.50
II	Supraacetabular	5.39	3.05
	Symphysial	NA ^1^	NA ^1^
	Ischial	4.96	4.13
III	Supraacetabular	3.01	6.33
	Symphysial	4.06	1.06
	Ischial	4.00	3.75
IV	Supraacetabular	2.34	2.42
	Symphysial	3.38	3.35
	Ischial	3.36	2.84
V	Supraacetabular	1.94	1.93
	Symphysial	4.75	5.86
	Ischial	4.59	2.52
VI	Supraacetabular	5.12	5.84
	Symphysial	5.75	7.46
	Ischial	1.58	2.63
VII	Supraacetabular	5.75	2.58
	Symphysial	5.72	4.04
	Ischial	3.05	4.56
VIII	Supraacetabular	3.80	3.72
	Symphysial	5.71	3.88
	Ischial	4.13	2.77
IX	Supraacetabular	0.66	2.25
	Symphysial	1.02	1.59
	Ischial	3.28	5.01
X	Supraacetabular	1.37	1.20
	Symphysial	5.77	6.17
	Ischial	3.99	5.42

^1^ Data not available for Case II (Symphysial osteotomy) due to structural failure of the phantom. The support broke completely during the symphysial osteotomy, compromising the pubic branch and preventing reliable analysis.

**Table 4:** Mean regional and overall angular deviations in osteotomies.

Osteotomy	FINAL Cut vs. PLANNED Cut Mean Angular Error (°)	FINAL Cut vs. PLACED PSI SUGGESTED Cut Mean Angular Error (°)
Supraacetabular	3.10	3.04
Symphysial	4.54	4.00
Ischial	3.63	3.61
Total	3.73	3.54
CI (95%) overall angular deviation	(1.79, 4.40)	(1.76, 4.32)

**Table 5:** Translation and orientation errors of the supraacetabular marker.

Marker	T_x_(mm)	T_y_ (mm)	T_z_ (mm)	R_x_ (°)	R_y_ (°)	R_z_ (°)
I	−1.31	1.74	−1.37	0.42	0.03	0.45
II	−2.53	−1.56	−0.97	2.51	0.23	−0.40
III	−2.13	0.31	−1.27	−0.30	−1.68	−1.11
IV	−0.56	0.05	−0.50	−0.34	−0.23	0.25
V	−1.28	0.58	0.29	0.00	0.00	0.00
VI	−0.54	1.77	−0.80	−1.28	0.14	2.09
VII	−1.12	0.02	−0.74	1.56	0.88	−3.34
VIII	0.99	2.32	−0.05	−1.09	1.49	0.09
IX	−1.06	1.18	−0.88	−1.30	−1.48	0.09
X	−2.26	0.99	−0.92	−1.50	−1.07	−0.48

**Table 6:** Mean values and standard deviations of time (in seconds) recorded for the placement and fixation of the PSIs, as well as the execution of osteotomies.

Time (s)	PTSupra	FTSupra	PTSymph	FTSymph	PTIsch	FTIsch	OTSupra	OTSypmh	OTIsch
Mean time	5.05	36.54	5.12	28.83	6.84	31.55	6.55	43.91	76.31
Deviation	(2.91)	(31.84)	(1.95)	(10.15)	(9.99)	(7.21)	(42.26)	(37.47)	(95.74)

Abbreviations: PTSupra, time for supraacetabular PSI placement; FTSupra, time for supraacetabular PSI fixation; PTSymph, time for symphysial PSI placement; FTSymph, time for symphysial PSI fixation; PTIsch, time for ischial PSI placement; FTIsch, time for ischial PSI fixation; OTSupra, time for supraacetabular osteotomy; OTSymph, time for symphysial osteotomy; and OTIsch, time for ischial osteotomy.

## Data Availability

Data will be provided upon request from the corresponding author.

## Abbreviations

The following abbreviations are used in this manuscript:

AR	Augmented Reality
ASA	Acrylonitrile Styrene Acrylate
CT	Computer Tomography
HMD	Head-Mounted Display
ICP	Iterative Closest Point
MRTK	Mixed Reality Toolkit
OTS	Optical Tracking Systems
PLA	Polylactic Acid
PSI	Patient-Specific Instrument
